# Chemistry and chemical biology tools contributing to the discovery and functional characterization of strigolactones

**DOI:** 10.3389/fpls.2025.1618437

**Published:** 2025-06-18

**Authors:** Qian Zhou, Changbin Niu, Liang Feng, Meixiu Dong, Xiaoxu Li, Bo Kong, Changsheng Li

**Affiliations:** ^1^ Hunan Research Center of the Basic Discipline for Cell Signaling, Hunan Provincial Key Laboratory of Plant Functional Genomics and Developmental Regulation, College of Biology, Hunan University, Changsha, China; ^2^ Beijing Life Science Academy, Beijing, China; ^3^ Tobacco Chemistry Research Institute of Technology Center, China Tobacco Hunan Industrial Co., Ltd., Changsha, China; ^4^ Yuelushan Laboratory, Changsha, China

**Keywords:** strigolactone, chemistry, chemical biology, biosynthesis, root exudate, synthetic biology

## Abstract

Strigolactones are a newly identified group of phytohormones that regulate plant growth and development and also act as communication signals in the rhizosphere. Beyond their well-known activity in stimulating parasitic weed germination, strigolactones function in regulating plant architecture, promoting symbiosis with arbuscular mycorrhizal fungi, and modulating responses to various environmental stresses. However, their low abundance, structural diversity, and instability have hindered comprehensive research and their practices. In this review, from the perspective of biological researcher, we summarize the powerful tools and strategies related to chemistry and chemical biology used in strigolactone area, covering analytical chemistry tools for isolation and structural elucidation, synthetic chemistry for structural elucidation and agricultural applications, chemical biology and biosynthetic strategies for functional characterization. Biosensors and probes used in monitoring strigolactone activity and signaling were also highlighted. Finally, we address current challenges and discuss future research perspectives, aiming to provoke more investigations on strigolactone biology and further boost their agricultural practices.

## 1 Introduction

**Figure 1:**
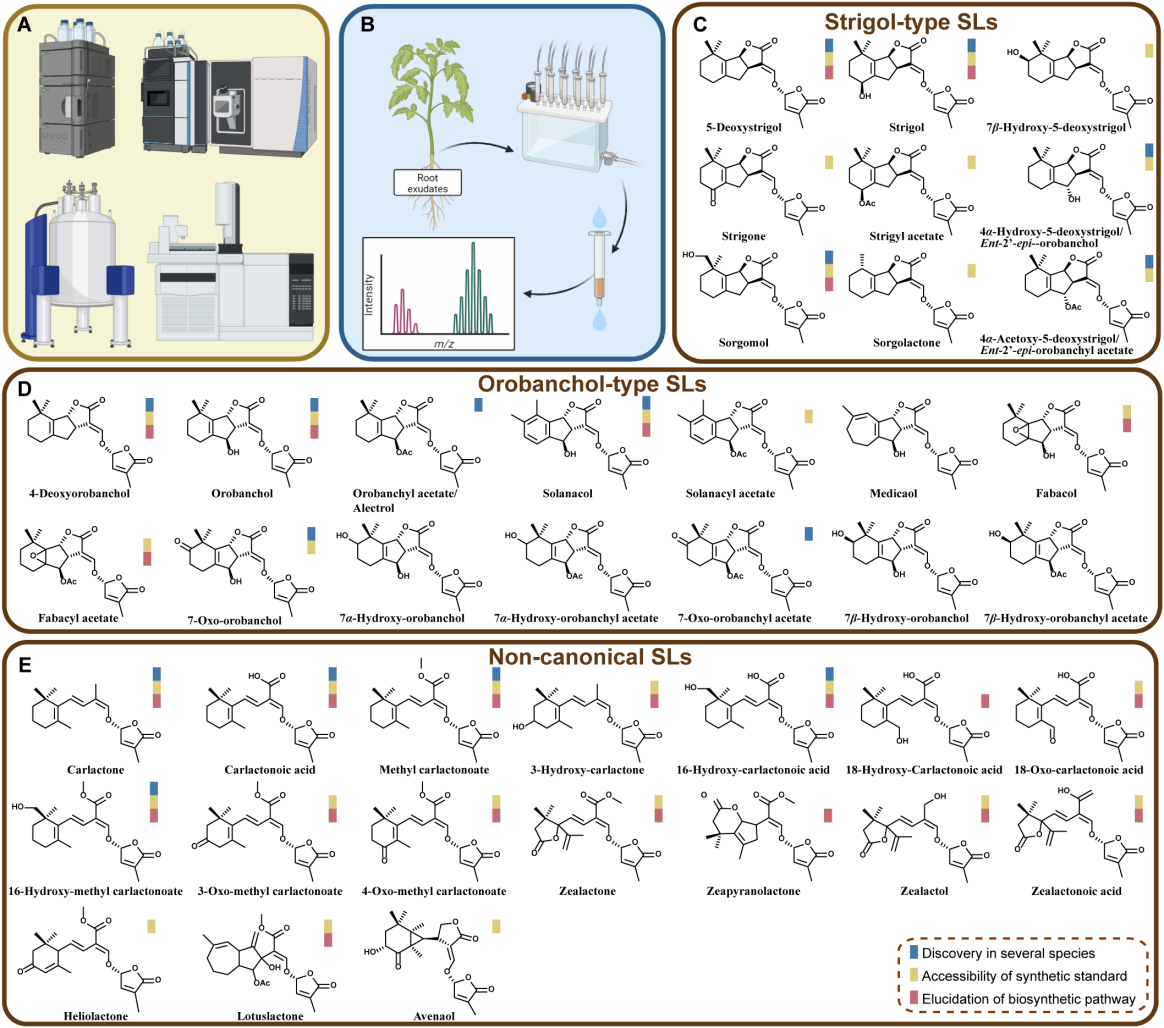
Analytical chemistry methods in advancing the identification of natural SLs. **(A)** Some main analytical chemistry instruments used in SL isolation and structural identification, including HPLC, LC-MS/MS, NMR, and GC-MS. **(B)** Scheme of workflow illustrating the extraction of root exudates, purification and detection. **(C-E)** Names and structures of natural SLs characterized from plants. On the basis of available literature, scope of their existence, the availability of synthetic standards and the progress of their biosynthesis pathways are indicated by three colored-boxes.

The history of strigolactones (SLs) research began with the isolation of strigol from cotton root exudates, the first identified natural SL (Cook et al., 1966). Over the years, more than 35 natural SLs have been identified from various plant species, which are mostly been detected from root exudates or roots (Guercio et al., 2023; Daignan-Fornier et al., 2024). Based on their structure characters, these SLs are derived carotenoid pathways and can be divided into two groups or three types (Yoneyama et al., 2018b; Guercio et al., 2023) (**Figure 1**). Canonical SLs, such as Strigol and Orobanchol (Cook et al., 1966; Mori et al., 1999; Al-Babili and Bouwmeester, 2015), are characterized by a tricyclic lactone structure comprising ABC-rings linked to a butenolide group (D-ring) through an enol-ether bridge. Non-canonical SLs, on the other hand, lack the complete structure of ABC rings but retain the conserved D-ring moiety, which is crucial for their biological activities (Zwanenburg et al., 2009; Kim et al., 2014; Ueno et al., 2014; Umehara et al., 2015; Charnikhova et al., 2017; Xie et al., 2019).

As mentioned, SLs were initially identified for their roles in stimulating the germination of parasitic weeds such as *Striga* and *Orobanche* (Cook et al., 1966; Parker, 2009; Westwood et al., 2010). Later in 2005, the positive biological function of SLs in promoting hyphal branching of arbuscular mycorrhizal fungi was uncovered, which further enhances nutrient uptake through symbiosis (Akiyama et al., 2005). Until 2008, SLs were recognized as key regulators of plant architecture by inhibiting shoot branching (Gomez-Roldan et al., 2008; Umehara et al., 2008). Later, more roles of SLs have been discovered, including responding to various biotic and abiotic stresses, by modulating plant growth and architecture (Brewer et al., 2013; Ruyter-Spira et al., 2013; Omoarelojie et al., 2019; Wu et al., 2022; Dun et al., 2023). For instance, in a recent study, role of SLs in safeguarding plants against abiotic stresses was uncovered, which is achieved by modulating stomatal activity, reducing transpirational water loss, enhancing nutrient uptake efficiency, and thereby strengthening overall plant resilience (Rhaman et al., 2024). Overall, these findings highlight SLs as an endogenous phytohormone and also signaling molecule in rhizosphere communications. The biosynthesis of SLs involves a core pathway starting from β-carotene and forming Carlactone (Matusova et al., 2005; Alder et al., 2012), which is catalyzed by three enzymes D27 (DWARF27), CCD7 (carotenoid cleavage dioxygenase 7), and CCD8 (carotenoid cleavage dioxygenase 8). After the production of carlactone, different structures of SLs can be biosynthesized by diversified branching pathways (Mashiguchi et al., 2020; Seto, 2023). In these steps, cytochrome P450 enzymes, methyltransferases, and other enzyme classes are involved in modifying the ABC-ring structures through a variety of reactions, including oxidations and methylations (Wu and Li, 2021; Yoda et al., 2021; Mashiguchi et al., 2022; Li et al., 2023; Kuijer et al., 2024; Li et al., 2024).

**Figure 2:**
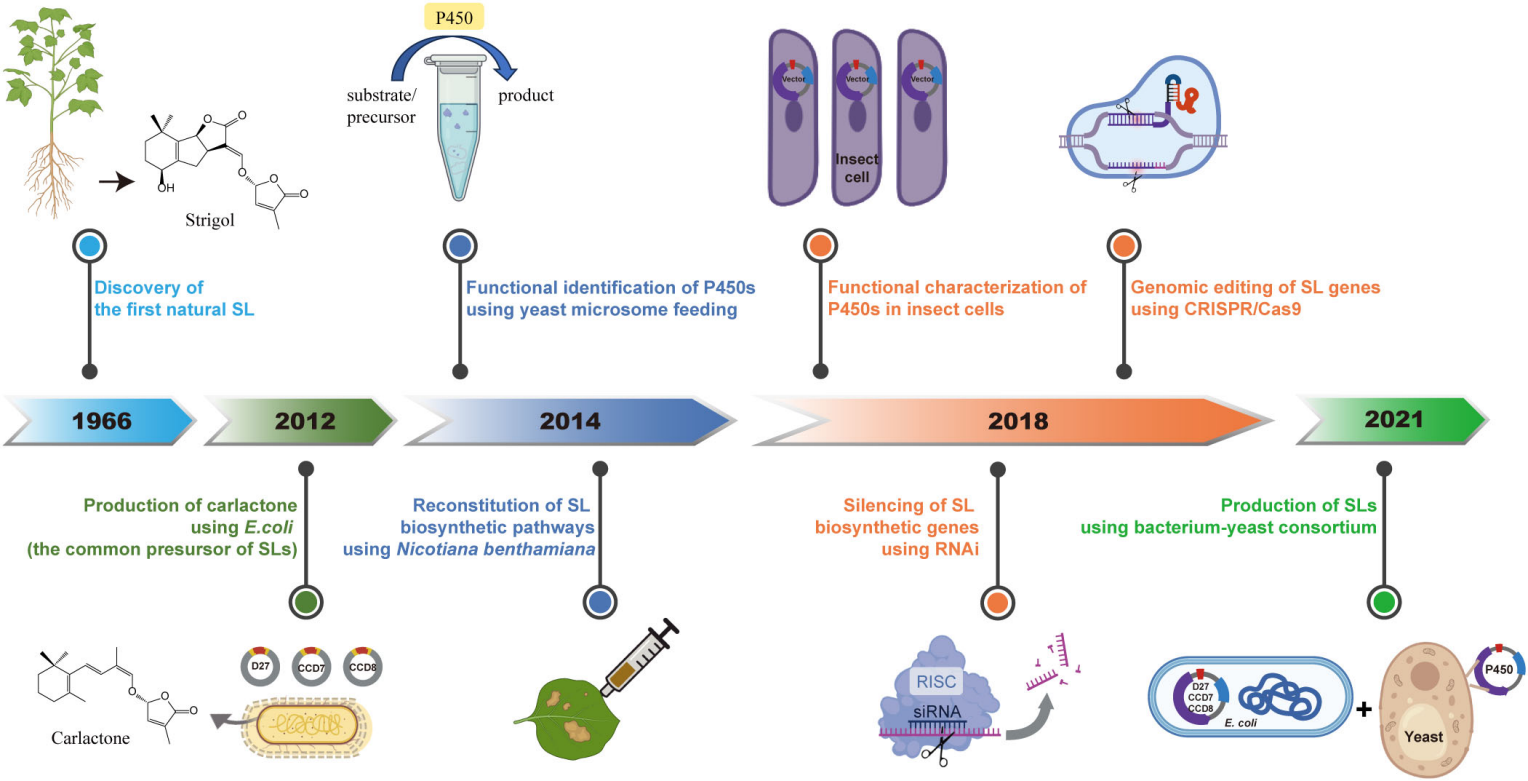
Milestones in SL research related to chemical biology and biosynthesis. This timeline illustrates a few key advances in SL research, from the discovery of Strigol in 1966, to modern engineering strategies. Highlights include the microbial production of Carlactone in *E. coli* in 2012, functional identification and reconstitution of SL biosynthetic enzymes in *Nicotiana benthamiana* in 2014, and the characterization of cytochrome P450s via yeast microsome feeding and insect cell systems. In 2018, RNAi-mediated silencing and CRISPR/Cas9-based genome editing enabled functional dissection of SL biosynthetic genes. More recently, a bacterium-yeast consortium has been established to produce SLs, representing a synthetic biology platform for scalable SL production.

In the research lines of SLs, advances in chemical and chemical biology techniques, such as the rapid development of mass-spectrometric (MS) techniques and Nuclear Magnetic Resonance Spectroscopy (NMR), have greatly contributed to the discovery of new SLs and the novel genes/enzymes in their biosynthetic pathways (Charnikhova et al., 2017; Floková et al., 2020; Rial et al., 2020; Xie et al., 2021; Lailheugue et al., 2023; Li et al., 2023) (**Figure 1**). In recent years, driven by interdisciplinary approaches including analytical chemistry tools and chemical biology strategies, the isolation and detection of SLs have become more simplified and efficient, facilitating the identification of new SLs and functional characterization of related genes (Zhang et al., 2014; Wu et al., 2021; Li et al., 2023, 2024; Zhou et al., 2025) (**Figure 2**). The development of organic synthesis and synthetic biology has enabled the relatively large-scale production of SLs and their analogs, among which GR24 is the most widely known and used one in functional studies of SLs (Krasylenko et al., 2021; Wu et al., 2021; Pyrzanowska-Banasiak et al., 2023; Tian et al., 2024; Zhou et al., 2025). This also promote the design and application of suicidal germination inducers in combatting parasitic weeds. The emergence of biosensors and reporter systems combined with chemical probes designed to target SL receptors or biosynthetic enzymes, have opened new opportunities for dissecting the molecular mechanisms underlying SL-mediated processes (Tsuchiya et al., 2015; Samodelov et al., 2016; Chesterfield et al., 2020; Germain et al., 2022; Song et al., 2022; Wang et al., 2022a). In this review, from the perspective of biological researcher, we summarize the tools and strategies related to chemistry and chemical biology utilized in SL research, highlighting recent advancements in analytical methods and biosensor development. By providing a comprehensive overview of the latest findings, this review aims to guide future research at enhancing plant resilience against biotic and abiotic stresses, by using knowledge of phytohormones and signaling molecules.

## 2 Analytical chemistry tools in SL identification

High-Performance Liquid Chromatography (HPLC) has been the traditional method for SL separation, typically using C18 reversed-phase columns with mobile phases composed of water and organic solvents like methanol or acetonitrile (Halouzka et al., 2020) (**Figure 1A**). Besides, Ultra-High-Performance Liquid Chromatography (UHPLC) has become popular due to its higher separation efficiency and shorter analysis time (Floková et al., 2020). Coupled with MS, UHPLC-MS/MS, particularly in multiple reaction monitoring (MRM) mode, is now widely used for identification and quantification of SLs (**Figure 1A**)., achieving even attomolar detection limits (Rial et al., 2020) and the discovery of several new SLs (Charnikhova et al., 2017; Halouzka et al., 2020; Karniel et al., 2024; Li et al., 2024). This method could provide rather high sensitivity and specificity for SL detection. Most of natural SLs are typically analyzed in positive ion mode, with transitions of sodium adduct ions [*M+H*]^+^ or [*M+Na*]^+^ being commonly monitored (Floková et al., 2020; Halouzka et al., 2020), while a few SLs can be preferably detected in negative ion mode, such as Carlactonoic acid (Floková et al., 2020). The application of stable isotope-labeled analogs (e.g., [^2^H_6_]-5-deoxystrigol and [^2^H_6_]-2′-*epi*-5-deoxystrigol) as internal standards helps the correct for variations during extraction and ionization efficiency (Floková et al., 2020). It is noteworthy that there are enantiomers for natural SLs and chiral column showed great capability in separation of these isomers (Wang et al., 2022b).

NMR is another powerful tool for determining the detailed structure of SLs, especially for confirming the presence of chiral centers and functional groups (**Figure 1A**). It is particularly useful for structural elucidation of newly discovered SLs (Ćavar et al., 2015; Charnikhova et al., 2017; Wang and Bouwmeester, 2018; Floková et al., 2020; Li et al., 2023; Nomura et al., 2023). In addition, modern ambient techniques such as Direct Analysis in Real Time (DART) and Desorption Electrospray Ionization (DESI) offer other options for SL detection, which is faster and only require a few sample preparation steps (Halouzka et al., 2020). These techniques hold promise for rapid SL identification but require optimization to address issues like sample shrinkage and matrix effects (Halouzka et al., 2020).

Solid-phase extraction (SPE) is a widely used sample preparation technique (Poole, 2003; Khatibi et al., 2021) (**Figure 1B**). The broad polarity range of SLs and their low abundance in plant related samples pose significant challenges for precise detection. Nevertheless, the application of SPE helps alleviate matrix effects, crucial for improving the accuracy and sensitivity of SL quantification. Although the culture system or sample collection methods can be diverse, SPE shows attractive ability to concentrate and purify SLs from complex and relatively large volumes of biological matrices (mainly from root exudates, root tissue extracts, etc.). For instance, columns, such as C18 and HLB, have shown great performance and been applied in SL extraction in multiple plant species (López-Ráez et al., 2008; Floková et al., 2020).

## 3 Chemistry boosts the discovery and function analysis of SLs

The development and application of these powerful chemical tools have advanced the discovery of natural SLs from different plants (**Figure 1C**). According to our collected information from literature, at least 40 natural forms of SLs have been found. As mentioned, Strigol, belonging to “Strigol-type”, was the first discovered SL (Cook et al., 1966). Later on, the pictures of “Strigol-type” and “Orobanchol-type” SLs had been expanded due to the discovery of more structures. During this process, scientists had dug more into the characteristics of these natural SLs’ structures and stereochemistry (Flematti et al., 2016; Xie, 2016; Wang and Bouwmeester, 2018; Yoneyama et al., 2018b). So far, all natural SLs pose 2’ *R* orientation in D ring, which is essential for their biological activities, on inducing the germination of parasitic weeds or inducing the hyphal branching of symbiotic arbuscular mycorrhizal fungi (AMF) (Zwanenburg et al., 2009; Akiyama et al., 2010; Boyer et al., 2012).

It is noteworthy that in recent years (after 2012) quite several new structures from “non-canonical” group have been uncovered (**Figure 1C**). For instance, several maize SLs, including Zealactone, Zeapyranolactone, Zealactol, and Zealactonoic acid, were identified from a variety of maize lines (Charnikhova et al., 2017; Xie et al., 2017; Charnikhova et al., 2018; Li et al., 2023). Besides, Avenaol, Lotuslactone, and Heliolactone were discovered from root exudates of *Helianthus annuus* (sunflower), *Lotus japonicus*, and *Avena strigosa*, respectively (Kim et al., 2014; Ueno et al., 2014; Xie et al., 2019). It can be also noticed that several research groups have found more and more derivatives of SL precursors (i.e., Carlactone, Carlactonoic acid, and Methyl carlactonate) (**Figure 1C**).

In most of the cases, the natural SLs were extracted and detected from root exudates or root tissues. However, very recent, 16-Hydroxy-carlactonoic acid (16-OH-CLA) was identified to be a product by the conversion of CYP722A, by the use of a microbial consortium expression system (Wu et al., 2021; Zhou et al., 2025) (detailed description of this method is present in next section and **Figure 2**). This form of SL and its derivative methyl 16-Hydroxycarlactonoate (16-OH-MeCLA) were detected only in the shoot tissues of several seed plants, including *Arabidopsis thaliana*, poplar (*Populus nigra* × *P. grandidentata*) and pepper (*Capsicum annuum*), plum (*Prunus mume*), and *Nelumbo nucifera*. This SL shows bioactivity in suppressing axillary shoot branching, in a manner dependent on SL signaling (Zhou et al., 2025). Possibly more natural structures will be characterized from other plant tissues and more biological functions of these SLs could be explored in the coming future.

After the first discovery of phytohormonal function of SLs (Gomez-Roldan et al., 2008), more and more analogs or mimics of natural SLs have been designed and synthesized, which are widely used in agriculture applications. These include GR24 (the most widely applied and the most famous one) (Akiyama et al., 2005; Gomez-Roldan et al., 2008), 4-Br debranone (4BD) (Fukui et al., 2013), MPs (Methyl phenlactonoates) (Jamil et al., 2018), Nijmegen-1 (Mwakaboko and Zwanenburg, 2016; Kountche et al., 2019; Jamil et al., 2022), 2NOD (2-nitrodebranone (Li et al., 2021) and other synthetic compounds. The scope of their practices could be roughly divided into germination stimulants of parasitic weeds (Suzuki et al., 2022), crop growth/architecture regulators (Jamil et al., 2020), helper-molecules for greater stress tolerance (Bhoi et al., 2021; Qi et al., 2024). For instance, exogenous application of GR24 in several species suggested the potential of this compound in increasing drought resistance (Cao et al., 2024; Dong et al., 2024; Ge et al., 2024; Shu et al., 2024). In future, along with our better understanding of the activities based on specific groups/subsections of natural SLs and other phytohormones, more targeted and accurate designing of active forms of synthetic analogs/mimics could be achieved.

## 4 Chemical biology and biosynthetic strategies in SL functional characterization

Although the first SL was discovered in 1960s (Cook et al., 1966), elucidating the biosynthesis and functions had been a big challenge. With the rapid advancement of science and technology, integration of chemical biology and biosynthetic methods has provided new opportunities for tackling these issues and broaden our knowledge of SLs’ nature. Here we summarize some key discovery of this process.

In 2012, researchers elucidated the biosynthetic pathway of the SL precursor, Carlactone, by transferring three SL biosynthetic genes into *Escherichia coli* and supplying appropriate substrates (Alder et al., 2012). This discovery laid a foundation for SL biosynthesis research, as Carlactone is considered as the common precursor of all natural SLs. Subsequently, in 2014, through yeast microsome feeding experiments and the reconstruction of the SL synthesis pathway in *Nicotiana benthamiana*, the catalytic activity and function of some MAX1s (belonging to cytochrome P450 711A family) was uncovered (Abe et al., 2014; Zhang et al., 2014). This first identification of P450 involved in SL biosynthesis has inspired investigation on other members, which plant scientists are stilling working on. In 2018, utilizing RNA silencing technology, the role of tomato SlMAX1 in SL biosynthesis and plant growth was characterized (Zhang et al., 2018). Additionally, CRISPR-Cas9 technology enabled the targeted editing of rice *CCD7* gene and analysis of its mutant showed the SL function in regulating plant height and tillering and further enhancing yield (Butt et al., 2018). Beyond yeast and plant expression system, the use of a baculovirus expression in insect cells has also confirmed the function of MAX1s across diverse plant species (Yoneyama et al., 2018a). These investigations provide a broader perspective on the biosynthesis and functional exploration of SLs.

The extremely low concentrations of SLs within plants have constrained both basic research and practical applications based on SLs. In 2021, a group developed a co-culture system of *E. coli* and yeast, establishing a microbial biosynthetic platform for the synthesis of various SLs (Wu et al., 2021). This innovative platform lays a solid foundation for the development of microbial production processes for SLs, marking a significant step toward their widespread application in agriculture and biotechnology.

## 5 Chemical probes and biosensors in SL activity and signaling research

Our understanding of SL signaling pathways has been significantly advanced through the development of chemical probes and biosensors. The classical probe, Yoshimulactone Green (YLG), identified ShHTLs as SL receptors in parasitic plant *Striga hermonthica*, with a Km value of 0.63 µM (Tsuchiya et al., 2015). Among the SLs tested in the competition hydrolysis activity assay, 5DS showed the strongest IC50 value of 0.44 µM, consistent with its higher activity in inducing *Striga* germination. Later, aryloxyacetyl piperazines were discovered as potential suicidal stimulants for parasitic plants at extremely low concentrations (10^-8^ to 10^-17^ M) (Wang et al., 2022a). Another group designed profluorescent SL Guillaume Clavé (GC) probes, with coumarin-based probes being highly bioactive in pea and a resorufin probe effective in moss, while YLG was less effective (Germain et al., 2022). These findings highlight the importance of SL probe specificity across different species. Another tool, genetically encoded biosensors for monitoring SL activity and signaling pathways, has been designed, among which StrigoQuant and Strigo-D2 are two notable examples (Samodelov et al., 2016; Song et al., 2022). StrigoQuant is designed to quantify SL activity and specificity. It employs a luciferase-based reporter system to detect the degradation of the SMXL6 protein, a key target in the SL signaling pathway. Among the tested SLs, 5-Deoxystrigol (5DS) was the most sensitive form, even at a low concentration of 100 fM, which is comparable to the activity observed in YLG-based assays. This provides a quantitative tool for studying SL activity *in plana*. Strigo-D2 is also based on SMXL6, which could monitor SL signaling patterns at cellular resolution. It was shown that different cell types respond to SLs with varying kinetics. Based on the SL receptors DAD2 from *Petunia hybrida* and HTL7 from *Striga hermonthica*, two fluorescent biosensors were constructed (Chesterfield et al., 2020). The sensitivity of both can reach nanomolar level, allowing direct detection of SLs *in vitro* and *in vivo*. Together, these biosensors provide valuable tools for studying SL signaling in various plant contexts, offering insights into the complex dynamics and specificities of SL activity.

## 6 Challenges and future directions

In summary, in the past decades, analytical chemistry tools such as UHPLC-MS/MS, SPE, and ambient MS techniques have significantly advanced the detection and identification of SLs (**Figure 1**). These methods provide the sensitivity and specificity required to study SLs in complex biological samples, paving the way for future research in plant biology and agriculture. The development of highly sensitive and specific analytical methods continues to be a focus in SL research. Combining UHPLC with high-resolution mass spectrometry (HR-MS) and optimizing sample preparation protocols will further enhance the detection and quantification of SLs and their derivatives in various plant species (Wu and Li, 2021; Zhou et al., 2025). Additionally, the application of ambient MS techniques for spatial profiling of SLs in different plant tissues is an emerging area with significant potential (Halouzka et al., 2020). Moreover, techniques for *in situ* detection of natural forms of SLs without complicated extraction and purification steps would be highly needed, although a few profluorescence probes and fluorescence-based biosensor have been developed for binding SL receptors (Tsuchiya et al., 2015; Samodelov et al., 2016; Chesterfield et al., 2020; Germain et al., 2022; Song et al., 2022; Wang et al., 2022a). We have to admit that this is quite challenging due to the unique characteristics of SLs, including their extremely low abundance, structural complexity, and relative instability.

As mentioned, several chemical biology has developed rapidly and boosted the discovery of SL biosynthetic pathways and also their biological functions (**Figure 2**). It could be noticed that the speed of discovering new enzymes/genes shows relatively slower tendency although many of the biosynthetic pathways have already been uncovered. Currently and in the coming future, with the emergence of omics and vast amount of biological big data linked with it, computational screening of putative structures and predication of candidate biosynthesis genes in phytohormone would provide us more and more accurate evidence. This could show guidance for wet-experimental designs and significantly increase the efficiency. Another trend is the combination of multiple disciplines in phytohormone research. For instance, crop populations offer us the genetic material foundation in searching new compounds; chemical tools help in standard synthesis, structural identification and mimics designing; chemical biology and synthetic biology reveal powerful ability in functional analysis and agronomic application. With all these chemistry tools, our understanding and practices based on it would be greatly enriched and expanded.
